# Lactose Intolerance versus Cow’s Milk Allergy in Infants: A Clinical Dilemma

**DOI:** 10.3390/nu16030414

**Published:** 2024-01-31

**Authors:** Andy Darma, Khadijah Rizky Sumitro, Juandy Jo, Nova Sitorus

**Affiliations:** 1 Department of Child Health, Dr. Soetomo General Academic Hospital, Surabaya 60286, Indonesia; khadijah.rizky.sumitro-2023@fk.unair.ac.id; 2 Department of Child Health, Faculty of Medicine, Universitas Airlangga, Surabaya 60132, Indonesia; 3 Department of Biology, Faculty of Science and Technology, Universitas Pelita Harapan, Tangerang 15811, Indonesia; juandy.jo@uph.edu; 4 Mochtar Riady Institute for Nanotechnology, Tangerang 15811, Indonesia; 5 Danone Specialized Nutrition Indonesia, Jakarta 12940, Indonesia; nova.sitorus@danone.com

**Keywords:** cow’s milk, protein, lactase, lactose intolerance, allergy

## Abstract

Due to its very early introduction, cow’s milk is one of the first foods that can cause adverse reactions in human beings. Lactose intolerance (LI) and cow’s milk allergy (CMA) are the most common adverse reactions to cow’s milk. While LI is due to insufficient small intestinal lactase activity and/or a large quantity of ingested lactose, CMA is an aberrant immune reaction to cow’s milk proteins, particularly casein or β-lactoglobulin. However, the clinical manifestations of LI and CMA, particularly their gastrointestinal signs and symptoms, are very similar, which might lead to misdiagnosis or delayed diagnosis as well as nutritional risks due to inappropriate dietary interventions or unnecessary dietary restriction. Formula-fed infants with LI should be treated with formula with reduced or no lactose, while those with CMA should be treated with formula containing extensive hydrolyzed cow’s milk protein or amino acids. This review is therefore written to assist clinicians to better understand the pathophysiologies of LI and CMA as well as to recognize the similarities and differences between clinical manifestations of LI and CMA.

## 1. Introduction

Cow’s milk is one of the most consumed foods worldwide and is used as a common substitute for human breast milk [1,2]. Cow’s milk is a complete food, which contains macronutrients (lactose; casein and whey proteins; triacylglycerols and cholesterol) as well as micronutrients (calcium, magnesium, sodium, potassium, phosphorus, iron, zinc, copper, selenium, and vitamins A, D, E and K) [3]. Unfortunately, cow’s milk can trigger an adverse reaction in human bodies due to its various components and different pathophysiologies [4,5].

The terms “intolerance” and “allergy” are commonly used synonymously when referring to adverse food reactions (AFRs) [1,6]. Lactose intolerance (LI) and cow’s milk allergy (CMA) are the most prevalent adverse reactions to cow’s milk, which are used interchangeably by many parents and healthcare professionals [1,6,7]. This confusion stems from the fact that both LI and CMA exert similar gastrointestinal manifestations [4,5,7,8]. The term “milk intolerance” is often incorrectly used to describe a wide spectrum of gastrointestinal symptoms that appear upon consumption of cow’s milk or cow’s milk-based formula and disappear when intake is terminated [9]. The fact that parents of children with CMA will substitute cow’s milk or cow’s milk-based formula with hydrolyzed protein-based or soy milk-based formula (which are free of lactose as well) further hinders attempts to differentiate subjects with CMA from the ones with LI [10].

An international survey among healthcare professionals reported that 61% of respondents either agreed or strongly agreed that they were confident in distinguishing CMA from LI in infants. However, this contradicts another finding that 23% of respondents either agreed or strongly agreed with the statement “primary lactose intolerance in infancy is common”, which is incorrect [11]. Confusion between CMA and LI among infants may lead to a delayed accurate diagnosis, as well as inappropriate dietary restriction/ intervention that might impose nutritional risks [3,4,6,11]. For example, it is common for infants with CMA to be prescribed lactose-free milk formula, even though they actually could tolerate lactose and use it as an energy source [12]. It has been reported that general practitioners in Northern Ireland have prescribed inappropriate medications and therapeutic formula to non-breastfed infants with underlying CMA. Anti-regurgitation and anti-colic products as well as lactose-free and partially hydrolyzed formulas (PHF) are more frequently prescribed than hypoallergenic formulas, such as extensively hydrolyzed formula (EHF) and amino acid-based formula (AAF), in infants diagnosed with CMA [13].

Therefore, an understanding of the diagnostic approaches of LI as well as CMA would reduce misdiagnosis and could assist parents in making logical choices regarding feeding their children [4,5,8]. This article aims to provide an overview of the clinical manifestations and diagnoses of LI and CMA as well as to highlight the mechanistic differences and management approaches of both diseases.

## 2. Lactose Intolerance

Lactose is the primary carbohydrate in human breast milk and cow’s milk [5,6,12]. Human breast milk contains approximately 7.5 g/100 mL of lactose, as compared to approximately 5 g/100 mL of lactose in cow’s milk and other mammalian milk [12,14,15]. Upon intake, lactose is hydrolyzed into monosaccharides (i.e., glucose and galactose) by an enzyme called lactase. The resulting monosaccharides are subsequently absorbed into the blood through the intestinal mucosa [9,12,16]. Lactase is expressed only in mature enterocytes, at least at 34 weeks of gestational age, and localized at the tips of the villi [2,16].

**Figure 1:**
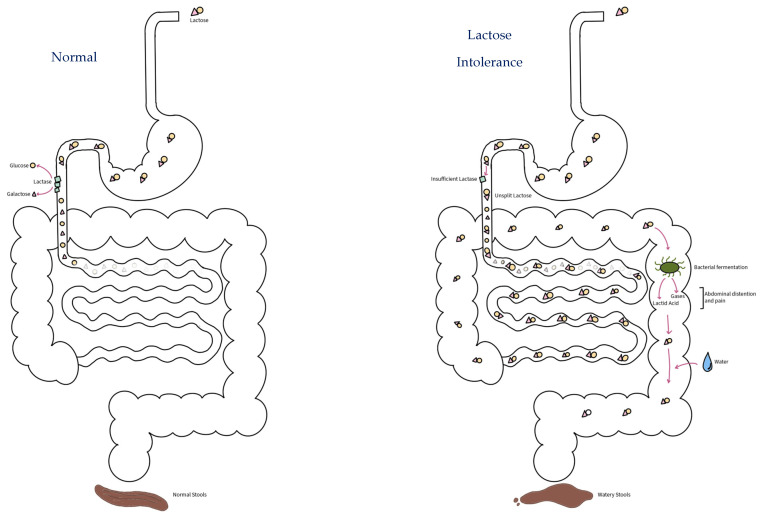
Mechanism of lactose intolerance. Lactose intolerance (**right**) occurs due to insufficient small intestinal lactase activity and/or a large quantity of ingested lactose. Its clinical manifestations are due to the undigested lactose being fermented by intestinal microbes leading to the release of high amounts of gases and lactic acids as well as drawing water into the intestinal lumen, causing abdominal pain, flatulence and diarrhea or watery stools. In a sufficient amount of lactase (**left**), lactose will be hydrolyzed into glucose and galactose and absorbed in the intestinal mucosa.

As shown in Figure 1, LI occurs due to the inability of the human body to digest and absorb dietary lactose (“lactose malabsorption”) due to low or absent activity of lactase (“lactase deficiency”) [12,16,17,18], and it is the most common carbohydrate intolerance during childhood [5]. Clinical manifestations of LI are indeed due to insufficient small intestinal lactase activity and/or a large quantity of ingested lactose, in which the undigested lactose would be fermented by intestinal microbes leading to the release of a high amount of gas as well as drawing water into the intestinal lumen, causing abdominal pain, flatulence, and diarrhea [1,19,20].

There are four types of LI based on the different causes, as discussed in the following.

### 2.1. Developmental Lactose Intolerance

Developmental lactose intolerance or neonatal lactose intolerance is observed in premature infants as lactase-expressing enterocytes in the small intestine only start to develop in the third trimester or at a gestational age of at least 34 weeks [5,16,21,22].

### 2.2. Congenital Lactose Intolerance

Congenital lactose intolerance or congenital/permanent lactase deficiency is due to a defect or mutation in the gene responsible for lactase synthesis, i.e., the lactase phlorizin hydrolase (*lph*) gene located in the human chromosome 2q21.3 [5,22]. The condition manifests as severe and intractable diarrhea during the neonatal period upon intake of human milk or lactose-containing milk formula. It is an inherited disease (probably an autosomal recessive disease) which is very rare and can be life threatening because of the risk of dehydration and electrolyte loss [1,2,6,16,23]. It is unknown yet whether heterozygote carriers of the *lph* gene are symptomatic or can even spontaneously resolve because of the plausibility that a wild-type lactase phlorizin hydrolase monomer might be able to dimerize with a mutated monomer in the endoplasmic reticulum to construct an active and transport-competent enzyme [24].

### 2.3. Primary Lactose Intolerance

Full-term infants are born with adequate lactase activity to digest lactose in milk, which is important for energy utilization [6,19]. Lactase activity physiologically reduces after 1 year of age, presumably due to a lower need for dairy products as a dietary source of energy at that stage [15]. However, among patients with primary lactose intolerance, their intestinal lactase expression decreases sharply from its peak at birth to less than 10% of the pre-weaning infantile level during childhood [14,16]. Primary lactose intolerance is indeed the most common type of LI and is referred to as adult-type hypolactasia, lactase non-persistence (LNP), primary lactase deficiency or hereditary lactase deficiency [16].

Primary lactose intolerance is also genetically determined. The geographical distribution and age at which lactase expression begins to decline differ by ethnicity, reflecting an underlying factor of genetic ancestry [4,12,25,26]. While children in Africa, Asia or Latin America may experience gastrointestinal manifestations between the age of 2 and 3 years old, children in Europe and North America typically do not develop gastrointestinal manifestations until later in childhood (5 to 6 years of age) or adolescence [4,12]. The age-related down-regulation of lactase activity is genetically programmed and a physiological phenomenon (as a result of the normal aging process or of dietary changes), which is slow, progressive in onset and irreversible [14,15,16,19,22,26].

### 2.4. Secondary Lactose Intolerance

Any pathological condition that damages the small intestine will lead to fewer mature enterocytes, which in return, reduces the expression of lactase. Those pathological conditions within the small intestine could cause secondary lactose intolerance [2,4,19]. Secondary lactose intolerance is usually transient (it is reversible once the epithelial lining has been repaired) and can occur at any age; although it is more common during infancy [7,16,19]. Secondary lactose intolerance can occur after acute gastroenteritis due to Rotavirus or Norovirus infection; persistent diarrhea caused by Giardiasis, Cryptosporidiosis and other parasites that infect the proximal small intestine; food allergy (including cow’s milk allergy); a short bowel which reduces the absorption’s surface; bacterial overgrowth in the small intestine; celiac disease; cystic fibrosis; Crohn’s disease; Acquired Immune Deficiency Syndrome (AIDS); as well as other immune-related enteropathies or radiation/chemotherapy-induced enteritis [4,15,16]. Of note, young infants with severe malnutrition could develop small intestinal atrophy that leads to a secondary lactase deficiency as well [16].

Among those contributing factors, the most common cause of secondary lactose intolerance in children is gastroenteritis [10,12]. It is relatively common among infants under two years of age, with the highest incidence during the first year of life, which was first reported by Burke et al. in 1965 [12,27,28]. The prevalence of secondary lactose intolerance upon Rotavirus infection was reported to be around 16.8% among children aged less than 5 years old in Spain [29] and around 11.2% among children aged less than 3 years old in Poland [30]. Norovirus-induced secondary lactose intolerance was reported in 3.4% of children aged less than 5 years old in Spain [29]. This relationship, however, was not observed in Malaysia, in which secondary lactose intolerance was only observed in 4 out of 393 children admitted with acute gastroenteritis [31]. This suggests that there is an unexplained factor yet underlying the association between gastroenteritis and secondary lactose intolerance.

## 3. Cow’s Milk Allergy

**Figure 2:**
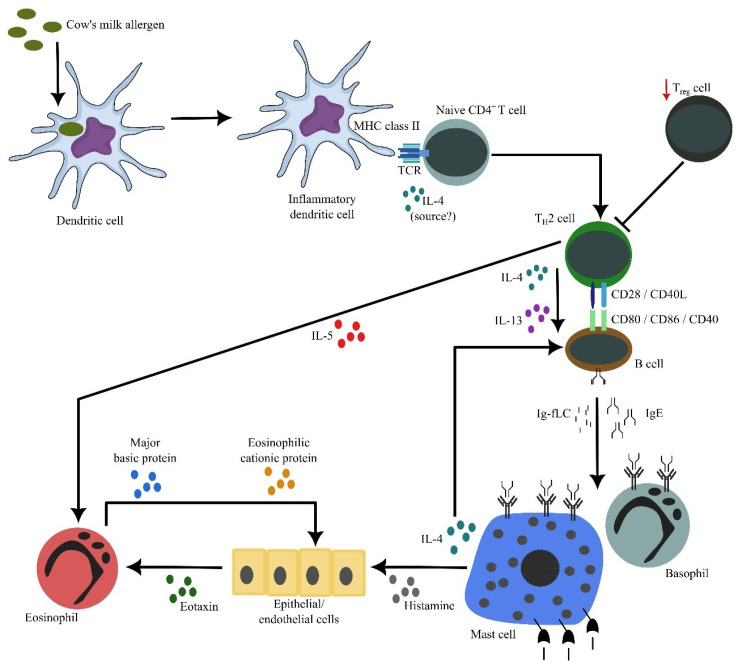
The cascade of inflammation in cow’s milk allergy. Allergens’ exposure to inflammatory dendritic cells allow these cells to process and present allergen-derived peptides to naïve CD4^+^ T cells. In the presence of IL-4 (from an unknown source), naïve CD4^+^ T cells differentiate into proallergic T_H2_ cells. Concurrently, it appears that there is an impairment of T_Reg_ cell frequency and/or activity, resulting in a lack of suppression of T_H2_ cell activity. Subsequently, T_H2_ cells will drive B cells, via cell contact as well as secreted IL-4 and IL-13, to undergo immunoglobulin class switch recombination, in which they eventually produce IgE. Along with antibody production, B cells also secrete significant amounts of κ and λ Ig-free light chains (Ig-fLCs). IgE and Ig-fLCs will then bind to mast cells and basophils, causing sensitization. Following subsequent exposure to allergens, cross-linking of surface-bound antibodies occurs, causing mast cells and basophils to degranulate and release their biologically active substances, including histamine, IL-4 and IL-5. Secreted IL-4 amplifies the differentiation between T_H2_ and IgE-producing B cells, while secreted IL-5 by T_H2_ cells causes accumulation and activation of eosinophils in the affected tissues. Similarly, histamine causes epithelial or endothelial cells to release eotaxin that attracts eosinophils into the tissues. Activated eosinophils release active substances, including major basic and eosinophilic cationic proteins that are toxic to the surrounding cells, contributing to further inflammation. This figure is reproduced with permission from reference [32].

CMA is an immune-mediated hypersensitivity reaction to cow’s milk protein that can be reproduced, as depicted in Figure 2. CMA may affect one or more organ systems and can be life threatening [1,6,8]. Cow’s milk is usually the first food that causes allergy, and CMA is the most common food allergy during the first year of life. Most infants developed clinical manifestations often within 1 week after the introduction of cow’s milk-based formula (95% of cases) [8,9,32]. The symptoms developed among 60% of infants when they started to be fed the cow’s milk-based formula [22]. Of note, perinatal sensitization to cow’s milk proteins can occur because mothers transfer the proteins from maternal circulation to the fetal circulation across the placenta or through breast milk during feeding [6].

Cow’s milk proteins comprise two major fractions: casein (76 to 86%) and whey (14 to 24%). The latter comprises β-lactoglobulin (7 to 12%), α-lactalbumin (2 to 5%), serum albumin (0.7 to 1.3%) and serum immunoglobulins (1.4 to 2.8%) [6]. β-lactoglobulin and casein are the most allergenic proteins; although all those proteins are postulated to be allergenic [1,8]. Cow’s milk proteins are digested by gastric and pancreatic proteolytic enzymes as well as absorbed by the intestinal mucosa. The resulting high-molecular-weight peptides, in combination with permeability in intestinal mucosa and genetic allergic predisposition, most likely play a part in allergic development upon exposure to cow’s milk [8,33].

CMA can be classified as immunoglobulin E (IgE)-mediated or “acute onset”, non-IgE-mediated (cell-mediated) or “delayed onset”, and mixed IgE- and non-IgE-mediated types [8,22,34,35]. As its symptoms usually appear within minutes to 2 h after consuming cow’s milk or cow’s milk-based formula, IgE-mediated CMA is easier to diagnose than non-IgE-mediated CMA. It is more challenging to diagnose non-IgE-mediated CMA because the time interval between ingestion and symptoms can vary from a few hours (2 to 48 h) to several days [5,36]. Factors that may influence the development of CMA are genetic predisposition (“atopy”), the allergenicity of the protein, the timing and frequency of cow’s milk exposure as well as premature weaning off breast milk [8,37]. Environmental factors such as early usage of antibiotics that alter the colonization of intestinal flora as well as the preterm birth might contribute to the risk of contracting CMA as well [33].

## 4. Different Mechanisms and Manifestations between Lactose Intolerance and Cow’s Milk Allergy

According to the European Academy of Allergology and Clinical Immunology (EAACI) nomenclature task force in 2001, adverse reactions to food are called food hypersensitivity and can be differentiated into (i) food allergy, when it is mediated by an immunologic mechanism, and (ii) non-allergic hypersensitivity, when it is not mediated by an immunologic mechanism [38,39]. The traditional term “food intolerance” could be referred to as “non-allergic food hypersensitivity”. This previous nomenclature proposed that “hypersensitivity” should be used as the umbrella term that covers all adverse food reactions and is defined as objectively reproducible symptoms or signs, initiated by an exposure to a defined stimulus at a dose tolerated by normal subjects [38]. However, in the latest nomenclature of allergic disease and hypersensitivity reaction by the EAACI in 2023, hypersensitivity refers to an undesirable, uncomfortable or damaging response that arises from a tissue cell dysfunction or immune system overreaction and is classified as an allergy or an autoimmune response [40].

Of note, LI and CMA are diseases caused by the same food source (i.e., cow’s milk), but they occur through different mechanisms (i.e., LI is a non-immune-based, enzymatic-imbalance disorder, while CMA is an immune-based disorder—food allergy) and are caused by different components of cow’s milk (i.e., carbohydrate and protein, respectively) [4,5,17,33]. Although the causes and mechanisms of LI and CMA are different, the clinical manifestations are similar, which hinders proper distinction between patients with either disease [4,5,6]. This might lead to misdiagnosis (under-diagnosis or over-diagnosis) or delayed diagnosis of either LI or CMA as well as nutritional risks due to inappropriate dietary interventions or unnecessary dietary restriction [4,5,7,12]. The clinical manifestations of LI and CMA are summarized in Table 1.

The clinical manifestations of LI originate in the gastrointestinal tract. Undigested lactose will pass rapidly into the colon as a consequence of the high osmolarity of the intraluminal disaccharide (i.e., lactose) and will be fermented to short-chain fatty acids and gas (i.e., hydrogen and methane) by colonic bacteria [17,18]. The undigested lactose will also attract water into the lumen as because of the compensatory capacity of the large bowel to handle an excess osmotic load [44]. Collectively, this will produce watery and acidic diarrhea, anal skin irritation, abdominal distention and vomiting. The severity of LI varies widely, depending on the quantity of ingested lactose relative to the intestinal lactase activity [1,8]. Extra-intestinal symptoms, such as headache, vertigo, memory impairment and lethargy, have been also described in up to 20% of people with carbohydrate intolerance [4]. LI can be suspected among infants who develop gastrointestinal symptoms shortly after ingesting cow’s milk. Diagnosis can be clinically settled by documenting the absence of signs and symptoms upon ingestion of lactose-free milk as well as the recurrence of LI due to the reintroduction of regular cow’s milk [1,5,44]. The hydrogen breath test (HBT) is currently considered as the method of choice for assessing primary and secondary lactose intolerance due to its high sensitivity and specificity, its simplicity and non-invasiveness, as well as its low cost. The HBT indeed has shown a sensitivity of 76–100% and a specificity of 90–100% [45]. The jejunal biopsy for measuring lactase activity remains the gold standard assay. However, it is mostly used for research purposes as it is very invasive, requires a special laboratory procedure and the cost is high. Of note, the results can vary from one site to another site due to the irregular distribution of lactase in the small intestine mucosa [1,6,37,45].

CMA presents with a non-specific and broad range of clinical manifestations, of which none are pathognomonic [1,2,6]. Children with CMA can present with a variety of signs and symptoms from two or more organ systems (i.e., cutaneous, gastrointestinal or respiratory system) which can be life threatening [6,8]. The most common cutaneous reactions are urticaria, atopic dermatitis, angioedema and contact rashes. Gastrointestinal reactions can manifest as nausea, vomiting (including hematemesis), colic, diarrhea (including occult and frank blood), enterocolitis, colitis, constipation and transient enteropathies. Respiratory reactions can include rhinoconjunctivitis, asthma, wheezing or laryngeal edema. CMA can cause a life-threatening anaphylactic reaction as well [8]. Among children with CMA, the clinical presentations could differ among IgE-mediated reactions, non-IgE-mediated reactions and mixed reactions [4]. Of note, gastrointestinal manifestations of non-IgE-mediated CMA are frequently, but incorrectly, labeled as signs and symptoms of intolerance [5,7].

Clinical manifestations of CMA should be improved following strict avoidance of cow’s milk without any intake of symptomatic medication, and they would recur upon the reintroduction of cow’s milk [6]. Elimination and reintroduction of cow’s milk and its derivatives are essential for diagnosing CMA. The short-term diagnostic elimination diet is carried out for 1–2 weeks in IgE-mediated and 2–4 weeks in non-IgE-mediated CMA. All sources of cow’s milk should be eliminated from infants diets for formula-fed infants and from the maternal diet for breastfed infants [36]. An oral food challenge for the reintroduction of cow’s milk can be performed as, either, an open food challenge (OpenFC), single-blind food challenge (SBFC) when only the patient is unaware of the challenged content, or double-blind placebo-controlled food challenge (DBPCFC) when both patient and staff are unaware of the challenged content [46].

The gold standard in the diagnosis of CMA is DBPCFC, yet this method is seldom performed in daily practice [8,36]. For practical and economic reasons, an elimination diet and reintroduction with an OpenFC is recommended in infants [36]. This notion was supported by findings from a study comparing OpenFC and DBPCFC in patients with positive food challenges with peanuts from 2001 to 2022, reporting that there was no difference in threshold values or the severity of symptoms, even when stratified for age groups (children and adults). In children, moderate and severe symptoms were experienced in 83% and 17% for OpenFC as well as 80% and 20% for DBPCFC, respectively. This study thus found that OpenFC was non-inferior to DBPCFC, if performed with strict objective stop criteria by trained staff [46]. Reintroducing cow’s milk through an oral food challenge needs to be performed under medical supervision for IgE-mediated and more severe forms of non-IgE-mediated CMA, such as food protein-induced enterocolitis syndrome (FPIES), while reintroducing cow’s milk can be performed at home (“home reintroduction”) for other forms of non-IgE-mediated CMA. In case of a doubtful/inconclusive oral food challenge and for scientific reasons, the DBPCFC is recommended [36].

Of note, a skin prick test or elevated cow’s milk protein-specific IgE can be useful if IgE-mediated CMA is suspected [5,36]. The different pathophysiologies between LI and CMA indicate that children with LI can tolerate cow’s milk proteins (but can only tolerate a small amount of or no lactose), while children with CMA are able to tolerate lactose (but cannot tolerate cow’s milk protein). However, CMA might cause severe enteropathy with secondary lactase deficiency. In those patients, there would be an overlap of gastrointestinal manifestations due to CMA and LI [4,5].

Regurgitation, vomiting, crying, fussiness, diarrhea and poor appetite have been reported in both LI and non-IgE-mediated CMA, which might also mimic pediatric functional gastrointestinal disorders (FGIDs), gastroesophageal reflux disease (GERD), eosinophilic esophagitis (EoE), anatomic abnormalities or metabolic and neurological diseases. While infantile colic and regurgitation generally occur in the first three to four months of life, natural resolution mostly occurs between the ages of four and five months for colic and six months or later for regurgitation. In cases where projectile vomiting is the predominant symptom or where regurgitation begins within the first two weeks of life or beyond six months of life, this is not a typical gastroesophageal reflux and might indicate an increased likelihood of secondary gastroesophageal reflux associated with anatomic abnormalities or CMA. However, regurgitation that occurs more than five times a day is the most specific symptom of GERD (specificity of 70.9%), but it has a poor positive predictive value (22%) [36,47].

## 5. Nutritional Management for Patients with Lactose Intolerance or Cow’s Milk Allergy

The primary treatment for AFRs is to eliminate the causative food from the diet [4]. Among breastfed infants with LI, breastfeeding should be promoted and maintained, and it should not be interrupted in infants with acute or prolonged diarrhea. Infants who were breastfed while suffering with diarrhea had a smaller loss and shorter disease duration than those whose breastfeeding was interrupted [15] Among formula-fed infants with LI, a diet with low or no lactose should be prescribed only when a true diagnosis of LI is achieved [4,5]. Lactose can be reintroduced after 1 to 2 months once the signs and symptoms have resolved and the small intestinal lactase activity has been restored [5]. Of note, the restricted diet is only necessary for a limited time to avoid the nutritional disadvantages of reduced intake of calcium and vitamins [4,5,48]. Treatment of the underlying disease in secondary lactose intolerance might restore the lactase level and improve clinical manifestations of LI [4,5].

In contrast, the management strategy for infants with CMA is to avoid cow’s milk proteins as strictly as possible [1,4,6], while strict elimination of cow’s milk protein from the maternal diet should be the first step for breastfed infants, and the use of EHF or even AAF, along with strict avoidance of cow’s milk, is the preferred strategy for formula-fed infants. Soy protein-based formula may be used to treat CMA in infants due to financial and cultural considerations, as well as better palatability. However, it is not recommended for infants less than 6 months old [41,49,50,51,52,53]. Highly purified lactose is well tolerated by infants with CMA, for whom lactose restriction is only warranted if an enteropathy with secondary lactase intolerance is present [12,51].

Of note, an international survey of 1663 healthcare professionals reported that 63% of respondents either agreed or strongly agreed that they understood the differences between various hypoallergenic and lactose-free formulas. Regarding secondary lactose intolerance, 44% of respondents recommended lactose avoidance for infants who contracted viral gastroenteritis, followed by cow’s milk allergy-induced enteropathy (36%). Regarding IgE-mediated CMA for formula-fed infants under 6 months of age, 59% of respondents selected EHF as the first-line treatment, with uncertainty among some respondents regarding prescribing lactose-free (29%) or lactose-containing formula (30%) [11]. The older-generation EHFs were typically free of lactose. However, lactose has been added to EHFs in recent years, as the presence of lactose can help to increase calcium absorption. The addition of lactose also slightly increases the sweetness of EHF, which improves its overall palatability and its acceptance by older infants [5].

## 6. Conclusions

Lactose intolerance (LI) is a non-immunologic reaction to cow’s milk carbohydrate, while cow’s milk allergy (CMA) is an immunologic reaction to cow’s milk protein. It is important to distinguish between these conditions because cow’s milk can induce adverse reactions through various components and different mechanisms. Knowing the pathophysiologies and recognizing the similarities and differences between signs and symptoms will be the main points for accurately diagnosing and treating infants with LI or CMA. Elimination of the causative component from the diet is the main treatment for both conditions. Formula-fed infants with LI should be treated with formula with a reduced- or no-lactose diet, while those with CMA should be treated with formula containing hydrolyzed cow’s milk protein or no cow’s milk protein. Of note, lactose restriction is only warranted among CMA patients with secondary lactose intolerance.

## Figures and Tables

**Table 1:** Clinical manifestations of lactose intolerance and cow’s milk allergy [1,4,5,7,8,12,16,33,41,42,43].

Signs and Symptoms	LI	CMA
Nausea and/or vomiting	±	±
Chronic diarrhea	++	±
Bloating or abdominal distention	+	±
Abdominal cramps/pain	+	±
Flatulence	+	±
Borborygmi	+	±
Blood and/or mucus in the stool	−	+
Rectal bleeding	−	+
Perianal rash and irritation	+	−
Failure to thrive or poor weight gain	−	+
Skin manifestation (e.g., eczema or urticaria)	−	+
Respiratory manifestation (e.g., wheezing or asthma)	−	+
Anaphylaxis	−	+ (if IgE-mediated)
Family history of atopic disease or food allergy	−	+

LI, lactose intolerance; CMA, cow’s milk allergy; −: no symptoms visible in patients; ±: symptoms may or may not be visible in patients; +: symptoms visible in patients; ++: symptoms are more frequently visible in patients. Note: Congenital lactose intolerance is rare. Primary lactose intolerance is uncommon among infants, but it is quite prevalent among children. There may be an overlap of gastrointestinal symptoms caused by CMA and LI.

## Data Availability

Data are contained within the article.

## References

[1] Bahna S.L. (2002). Cow’s milk allergy versus cow milk intolerance. Ann. Allergy Asthma Immunol..

[2] Fox A.T., Thomson M. (2007). Adverse reactions to cow’s milk. Pediatr. Child Health.

[3] Antunes I.C., Bexiga R., Pinto C., Roseiro L.C., Quaresma M.A.G. (2023). Cow’s milk in human nutrition and the emergence of plant-based milk alternatives. Foods.

[4] Di Costanzo M., Berni Canani R. (2018). Lactose intolerance: Common misunderstandings. Ann. Nutr. Metab..

[5] Di Costanzo M., Biasucci G., Maddalena Y., Scala C.D., Caro C.D., Calignano A., Canani R.B. (2021). Lactose intolerance in pediatric patients and common misunderstandings about cow’s milk allergy. Pediatr. Ann..

[6] Bahna S.L. (1996). Is it milk allergy or lactose intolerance?. Immunol. Allergy Clin. N. Am..

[7] Walsh J., Meyer R., Shah N., Quekett J., Fox A.T. (2016). Differentiating milk allergy (IgE and non-IgE mediated) from lactose intolerance: Understanding the underlying mechanisms and presentations. Br. J. Gen. Pract..

[8] Wilson J. (2005). Milk intolerance: Lactose intolerance and cow’s milk protein allergy. Newborn Infant Nurs. Rev..

[9] Kaskous K. (2021). Cow’s milk consumption and risk of disease. Emir. J. Food Agric..

[10] Gracey M., Burke V. (1973). Sugar-induced diarrhoea in children. Arch. Dis. Child..

[11] Madrazo J.A., Alrefaee F., Chakrabarty A., de Leon J.C., Geng L., Gong S., Heine R.G., Järvi A., Ngamphaiboon J., Ong C. (2022). International cross-sectional survey among healthcare professionals on the management of cow’s milk protein allergy and lactose intolerance in infants and children. Pediatr. Gastroenterol. Hepatol. Nutr..

[12] Heine R.G., Alrefaee F., Bachina P., de Leon J.C., Geng L., Gong S., Madrazo J.A., Ngamphaiboon J., Ong C., Rogacion J.M. (2017). Lactose intolerance and gastrointestinal cow’s milk allergy in infants and children—Common misconceptions revisited. World Allergy Organ. J..

[13] Wauters L., Brown T., Venter C., Dziubak R., Meyer R., Brogan B., Walsh J., Fox A.T., Shah N. (2016). Cow’s milk allergy prescribing is influenced by regional and national guidance. J. Pediatr. Gastroenterol. Nutr..

[14] Vandenplas Y. (2015). Lactose intolerance. Asia Pac. J. Clin. Nutr..

[15] Toca M.D.C., Fernández A., Orsi M., Tabacco O., Vinderola G. (2022). Lactose intolerance: Myths and facts. An update. Arch. Argent. Pediatr..

[16] Heyman M.B. (2006). Lactose intolerance in infants, children, and adolescents. Pediatrics.

[17] ElShafie A.M., Shaheen H.M., Ebrahim E.S.A.E.B. (2015). Lactose intolerance among pediatrics: Systematic review. Menoufia Med. J..

[18] Wilt T.J., Shaukat A., Shamliyan T., Taylor B.C., MacDonald R., Tacklind J., Rutks I., Schwarzenberg S.J., Kane R.L., Levitt M. (2010). Lactose intolerance and health. Evid. Rep. Technol. Assess. (Full Rep.).

[19] Harvey L., Ludwig T., Hou A.Q., Hock Q.S., Tan M.L., Osatakul S., Bindels J., Muhardi L. (2018). Prevalence, cause and diagnosis of lactose intolerance in children aged 1–5 years: A systematic review of 1995-2015 literature. Asia Pac. J. Clin. Nutr..

[20] Gremse D.A., Greer A.S., Vacik J., DiPalma J.A. (2003). Abdominal pain associated with lactose ingestion in children with lactose intolerance. Clin. Pediatr..

[21] Antonowicz I., Lebenthal E. (1977). Developmental pattern of small intestinal enterokinase and disaccharidase activities in the human fetus. Gastroenterology.

[22] Al-Beltagi M., Saeed N.K., Bediwy A.S., Elbeltagi R. (2022). Cow’s milk-induced gastrointestinal disorders: From infancy to adulthood. World J. Clin. Pediatr..

[23] Diekmann L., Pfeiffer K., Naim H.Y. (2015). Congenital lactose intolerance is triggered by severe mutations on both alleles of the lactase gene. BMC Gastroenterol..

[24] Wanes D., Husein D.M., Naim H.Y. (2019). Congenital lactase deficiency: Mutations, functional and biochemical implications, and future perspectives. Nutrients.

[25] Misselwitz B., Butter M., Verbeke K., Fox M.R. (2019). Update on lactose malabsorption and intolerance: Pathogenesis, diagnosis and clinical management. Gut.

[26] Anguita-Ruiz A., Aguilera C.M., Gil Á. (2020). Genetics of lactose intolerance: An updated review and online interactive world maps of phenotype and genotype frequencies. Nutrients.

[27] Kumar V., Chandrasekaran R., Bhaskar R. (1977). Carbohydrate intolerance associated with acute gastroenteritis: A prospective study of 90 well-nourished Indian infants. Clin. Pediatr..

[28] Burke V., Kerry K.R., Anderson C.M. (1965). The relationship of dietary lactose to refractory diarrhoea in infancy. Aust. Paediatr. J..

[29] Gonzalez-Galan V., Sánchez-Fauqier A., Obando I., Montero V., Fernandez M., Torres M.J., Neth O., Aznar-Martin J. (2011). High prevalence of community-acquired norovirus gastroenteritis among hospitalized children: A prospective study. Clin. Microbiol. Infect..

[30] Szajewska H., Kantecki M., Albrecht P., Antoniewicz J. (1997). Carbohydrate intolerance after acute gastroenteritis—A disappearing problem in Polish children. Acta Paediatr..

[31] Poo M.I., Paeds M., Lee W.S. (2007). Admission to hospital with childhood acute gastroenteritis in Kuala Lumpur Malaysia. Med. J. Malays..

[32] Høst A. (2002). Frequency of cow’s milk allergy in childhood. Ann. Allergy Asthma Immunol..

[33] Rangel A.H.D.N., Sales D.C., Urbano S.A., Galvão J.G.B., Neto J.C.D.A., Macêdo C.D.S. (2016). Lactose intolerance and cow’s milk protein allergy. Food Sci. Technol..

[34] Zepeda-Ortega B., Goh A., Xepapadaki P., Sprikkelman A., Nicolaou N., Hernandez R.E.H., Latiff A.H.A., Yat M.T., Diab M., Hussaini B.A. (2021). Strategies and future opportunities for the prevention, diagnosis, and management of cow milk allergy. Front. Immunol..

[35] Jo J., Garssen J., Knippels L., Sandalova E. (2014). Role of cellular immunity in cow’s milk allergy: Pathogenesis, tolerance induction, and beyond. Mediat. Inflamm..

[36] Meyer R., Venter C., Bognanni A., Szajewska H., Shamir R., Nowak-Wegrzyn A., Fiocchi A., Vandenplas Y., WAO DRACMA Guideline Group (2023). World Allergy Organization (WAO) Diagnosis and Rationale for Action against Cow’s Milk Allergy (DRACMA) Guideline update—VII—Milk elimination and reintroduction in the diagnostic process of cow’s milk allergy. World Allergy Organ. J..

[37] Da Silva P.H.F., Oliveira V.C.D., Perin L.M. (2018). Cow’s milk protein allergy and lactose intolerance. Raw Milk: Balance between Hazards and Benefits.

[38] Johansson S.G.O., Hourihane J.O.B., Bousquet J., Bruijnzeel-Koomen C., Dreborg S., Haahtela T., Kowalski M.L., Mygind N., Ring J., van Cauwenberge P. (2001). A revised nomenclature for allergy. An EAACI position statement from the EAACI nomenclature task force. Allergy.

[39] Johansson S.G.O., Bieber T., Dahl R., Friedmann P.S., Lanier B.Q., Lockey R.F., Motala C., Martell J.A.O., Platts-Mills T.A.E., Ring J. (2004). Revised nomenclature for allergy for global use: Report of the Nomenclature Review Committee of the World Allergy Organization, October 2003. J. Allergy Clin. Immunol..

[40] Jutel M., Agache I., Zemelka-Wiacek M., Akdis M., Chivato T., Del Giacco S., Gajdanowicz P., Gracia I.E., Klimek L., Lauerma A. (2023). Nomenclature of allergic diseases and hypersensitivity reactions: Adapted to modern needs: An EAACI position paper. Allergy.

[41] Vandenplas Y., Koletzko S., Isolauri E., Hill D., Oranje A.P., Brueton M., Staiano A., Dupont C. (2007). Guidelines for the diagnosis and management of cow’s milk protein allergy in infants. Arch. Dis. Child..

[42] Matthews S.B., Waud J.P., Roberts A.G., Campbell A.K. (2005). Systemic lactose intolerance: A new perspective on an old problem. Postgrad. Med. J..

[43] Heuschkel R.B., Gottrand F., Devarajan K., Poole H., Callan J., Dias J.A., Karkelis S., Papadopoulou A., Husby S., Ruemmele F. (2015). ESPGHAN position paper on management of percutaneous endoscopic gastrostomy in children and adolescents. J. Pediatr. Gastroenterol. Nutr..

[44] Hull D., Bartrop R.W. (1973). Transient lactose intolerance in infancy. Arch. Dis. Child..

[45] Fassio F., Facioni M.S., Guagnini F. (2018). Lactose maldigestion, malabsorption, and intolerance: A comprehensive review with a focus on current management and future perspectives. Nutrients.

[46] Jessen F.B., Mortz C.G., Eller E., Gudichsen J.H., Baekdal E.A., Bindslev-Jensen C. (2023). A comparison of double-blind, placebo-controlled food challenge and open food challenge. Allergy Eur. J. Allergy Clin. Immunol..

[47] Salvatore S., Agosti M., Baldassarre M.E., D’Auria E., Pensabene L., Nosetti L., Vandenplas Y. (2021). Cow’s milk allergy or gastroesophageal reflux disease—Can we solve the dilemma in infants?. Nutrients.

[48] Usai-Satta P., Scarpa M., Oppia F., Cabras F. (2012). Lactose malabsorption and intolerance: What should be the best clinical management?. World J. Gastrointest. Pharmacol. Ther..

[49] Koletzko S., Niggemann B., Arato A., Dias J.A., Heuschkel R., Husby S., Mearin M.L., Papadopoulou A., Ruemmele F.M., Staiano A. (2012). Diagnostic approach and management of cow’s-milk protein allergy in infants and children: ESPGHAN GI Committee practical guidelines. J. Pediatr. Gastroenterol. Nutr..

[50] Vandenplas Y., Alarcon P., Alliet P., De Greef E., De Ronne N., Hoffman I., Winckel M.V., Hauser B. (2015). Algorithms for managing infant constipation, colic, regurgitation and cow’s milk allergy in formula-fed infants. Acta Paediatr..

[51] Espín Jaime B., Díaz Martín J.J., Blesa Baviera L.C., Claver Monzón Á., Hernández A.H., Burriel J.I.G., Mérida M.J.G., Fernández C.P., Rodríguez C.C., Riechmann E.R. (2019). Non-IgE-mediated cow’s milk allergy: Consensus document of the Spanish Society of Paediatric Gastroenterology, Hepatology, and Nutrition (SEGHNP), the Spanish Association of Paediatric Primary Care (AEPAP), the Spanish Society of Extra-hospital Paediatric. An. Pediatr. (Engl. Ed.).

[52] Toca M.C., Morais M.B., Vázquez-Frias R., Becker-Cuevas D.J., Boggio-Marzet C.G., Delgado-Carbajal L., Higuera-Carrillo M.M., Ladino L., Marchisone S., Messere G.C. (2022). Consensus on the diagnosis and treatment of cow’s milk protein allergy of the Latin American Society for Pediatric Gastroenterology, Hepatology and Nutrition. Rev. Gastroenterol. Méx. (Engl. Ed.).

[53] Vandenplas Y., Broekaert I., Domellöf M., Indrio F., Lapillonne A., Pienar C., Ribes-Koninckx C., Shamir R., Szajewska H., Thapar N. (2023). An ESPGHAN position paper on the diagnosis, management and prevention of cow’s milk allergy. J. Pediatr. Gastroenterol. Nutr..

